# Fusion protein analysis reveals the precise regulation between Hsp70 and Hsp100 during protein disaggregation

**DOI:** 10.1038/s41598-017-08917-8

**Published:** 2017-08-17

**Authors:** Sayaka Hayashi, Yosuke Nakazaki, Kei Kagii, Hiromi Imamura, Yo-hei Watanabe

**Affiliations:** 1 Department of Biology, Faculty of Science and Engineering, Konan University, Kobe, Japan; 2 Department of Functional Biology, Graduate School of Biostudies, Kyoto University, Kyoto, Japan; 3 Institute for Integrative Neurobiology, Konan University, Kobe, Japan

## Abstract

ClpB, a bacterial Hsp100, is a ring-shaped AAA+ chaperone that can reactivate aggregated proteins in cooperation with DnaK, a bacterial Hsp70, and its co-factors. ClpB subunits comprise two AAA+ modules with an interstitial rod-shaped M-domain. The M-domain regulates ClpB ATPase activity and interacts directly with the DnaK nucleotide-binding domain (NBD). Here, to clarify how these functions contribute to the disaggregation process, we constructed ClpB, DnaK, and aggregated YFP fusion proteins in various combinations. Notably, i) DnaK activates ClpB only when the DnaK substrate-binding domain (SBD) is in the closed conformation, affording high DnaK-peptide affinity; ii) although NBD alone can activate ClpB, SBD is required for disaggregation; and iii) tethering aggregated proteins to the activated ClpB obviates SBD requirements. These results indicate that DnaK activates ClpB only when the SBD tightly holds aggregated proteins adjacent to ClpB for effective disaggregation.

## Introduction

Reactivation of damaged proteins is an important process for the survival of cells under stress conditions^1, 2^. DnaK (bacterial Hsp70) and ClpB (bacterial Hsp100) constitute a chaperone system that can rescue the aggregated proteins^3–7^ and play an important role for the thermotolerance of bacterial and yeast cells^8, 9^.

DnaK consists of a nucleotide-binding domain (NBD) as well as a substrate-binding domain (SBD) that is subdivided into a β-sandwich substrate-binding core (SBDβ) and an α-helical lid (SBDα)^10, 11^. The NBD binds and hydrolyses ATP and controls the peptide-binding property of the SBD. In the ATP bound state, SBDα detaches SBDβ and the substrate-binding site becomes exposed to the solvent^12, 13^. In such open form, the SBD rapidly binds and releases denatured proteins with relatively low affinity^14, 15^. Conversely, in the ADP bound state, SBDα covers the substrate-binding site (termed the closed form)^16^, resulting in slow rates of peptide binding and release and high affinity^14, 15^. DnaJ, a co-chaperone of DnaK, also individually binds denatured proteins and stimulates the ATPase activity of DnaK^17^ whereas GrpE, another co-chaperone, accelerates the ADP/ATP exchange of DnaK^17^. Although, DnaK, DnaJ, and GrpE can assist with protein folding mainly by preventing the aggregation formation, their disaggregation activity is marginal^18^.

ClpB is a member of the ATPases associated with diverse cellular activities (AAA+proteins) superfamily^19, 20^ and consists of an N-terminal domain (ND), an M-domain (MD), and two AAA+ modules (AAA1 and AAA2)^21^. Each AAA+ module binds and hydrolyses ATP, and causes structural changes required for protein disaggregation^22–25^. Similar to other members of the AAA+ protein family, ClpB forms a ring shaped hexamer^21, 26–28^. The ring core consists of two tiered-rings: an AAA1-ring and an AAA2-ring, having central pores through which the substrate proteins are threaded^1, 29^. According to the recent cryo-EM structure of Hsp104, a yeast homologue of ClpB, a spiral structure in which these two AAA rings are sequentially connected was also proposed^30^. The globular ND of each subunit is tethered to the edge of the AAA1-ring and contributes to binding and processing of the substrate proteins^27, 31–36^. The rod-shaped MDs surround the AAA1-ring and are thought to act as modulators of ClpB^28, 37^. Notably, some mutations in MD and its surrounding region of ClpB were found to drastically change the ATPase and/or chaperone activities^28, 29, 38–40^. For example, the E432A mutant of *Escherichia coli* ClpB (*E*ClpB) resulted in the loss of chaperone activity and thus was termed a repressed mutant. Conversely, the Y503D mutant of *E*ClpB showed vastly enhanced ATPase and unfolding activity and was termed a hyperactive mutant. According to the cryo-EM analysis, MDs of the E432A and Y503D mutants of *E*ClpB formed different conformations, horizontal and tilted, respectively^28^. In the horizontal form, the MDs of neighboring subunits interact with each other and coil around the AAA1 ring whereas in the tilted form, the MD-MD interactions are relieved.

Recently, direct interaction between the NBD of DnaK and MD of ClpB was shown via NMR study^41^. The DnaK-dependent stimulation of ClpB ATPase activity was also found, although the stimulation required extremely high concentrations of DnaK and ClpB. Based on these results together with other related observations, the following model for the cooperation between DnaK and ClpB was proposed: i) DnaK binds aggregated protein; ii) ClpB binds DnaK through their respective MD and NBD, stimulating the ClpB ATPase activity; iii) DnaK transfers the aggregated proteins to the activated ClpB; and iv) ClpB threads the aggregated proteins through its central pore to disaggregate them. Although the model is attractive and plausible, there are some missing links. For example, it is not clear whether the ATPase activity activated by DnaK is coupled to protein disaggregation, how the ATPase cycle of DnaK correlates to the interaction between chaperones, and how the DnaK transfers the aggregated proteins to ClpB in step iii. To fully understand the chaperone machinery, details of the functional interactions between DnaK and ClpB, including the above questions, should be clarified. However, precise investigation of these issues has been difficult primarily owing to the low affinity between DnaK and ClpB (*K*_d_ ≈ 25 µM)^41^.

To overcome this difficulty, in this study we prepared several variations of DnaK and ClpB fusion proteins and tested their activities. We found that the NBD of DnaK could stimulate ClpB ATPase activities only when the SBD was omitted or was in the closed form. Moreover, the properties of these fusion proteins were compared with MD mutants possessing enhanced ATPase activities (hyperactive mutants). Both the hyperactive mutants and the fusion proteins could reactivate the aggregated proteins only when the aggregation was placed close to the chaperone. These results disclosed the regulatory mechanisms of the interaction between DnaK and ClpB during the protein disaggregation reaction.

## Results

### Characterization of *Thermus thermophilus* DnaK (*T*DnaK) mutants

**Figure 1:**
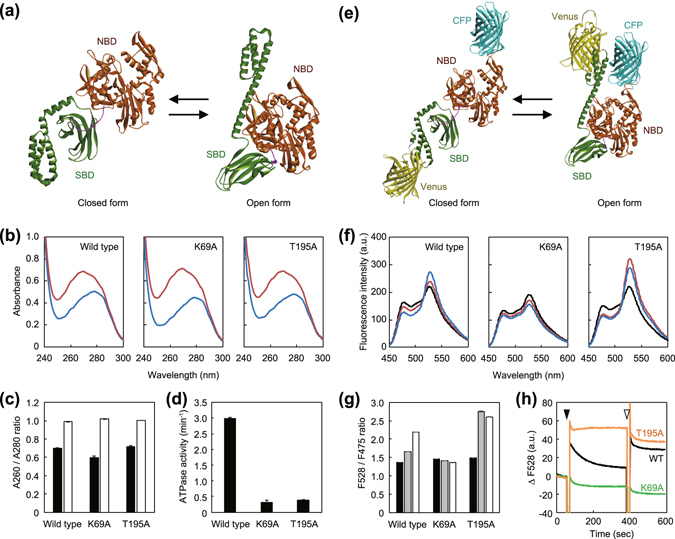
Characterization of K69A and T195A mutants of *T*DnaK. (**a**) Structural models of *T*DnaK constructed by homology modeling using DiscoveryStudio 4.5 software. *E*DnaK in the closed (PDBid:2KHO)^16^ and open (PDBid:4B9Q)^12^ forms were used as templates. The NBD and the SBD are colored by brown and green, respectively. (**b**) *T*DnaK and its mutants were subjected to HPLC size exclusion chromatography (SEC) in the presence of 10 mM EDTA to remove bound nucleotides and their UV spectra were measured (blue line). The nucleotide-depleted *T*DnaKs were incubated with 3 mM ATP and subjected to SEC to remove unbound nucleotides. The spectra of the eluted proteins were also measured (red line). (**c**) The A_260_/A_280_ ratio of the nucleotide-depleted (filled bar) and ATP-treated (open bar) *T*DnaKs are shown. (**d**) ATPase activities of *T*DnaK and its mutants (2.5 μM) measured in the presence of 5.0 μM *T*DnaJ, 5.0 μM *T*GrpE, and 3 mM ATP are shown. (**e**) Structural models of *T*K-FRET. The CFP and the Venus portions are colored by cyan and yellow, respectively. (**f**) *T*K-FRET and its mutants were treated as in b) and the fluorescence spectra of the nucleotide-depleted proteins in the absence (black line) and the presence of 2 mM ATP (red line), or the presence of 2 mM ATP and 1.0 μM *T*GrpE (blue line) were measured. (**g**) The F_528_/F_475_ ratio of the nucleotide-depleted *T*K-FRETs in the absence (filled bar) and the presence of 2 mM ATP (gray bar), or the presence of 2 mM ATP and 1.0 μM *T*GrpE (open bar) are shown. (**h**) Changes of F_528_ of the nucleotide-depleted *T*K-FRETs; wild-type (black), K69A (green), and T195A (orange) mutants are shown. The black and white arrows indicated the time points at which the ATP and *T*GrpE were added, respectively. (**f**)–(**h**) Excitation wavelength was 435 nm. (**c**,**d** and **g**) Error bars represent standard deviations of three or more independent measurements.

Recently, the structure of the ATP-bound open form of *E. coli* DnaK (*E*DnaK) was solved and a model for the ATPase-cycle dependent conformational changes was proposed (Fig. 1a)^12, 13^. In this model, ATP binding to the NBD induces the conformational change of DnaK to the open form, whereas ATP hydrolysis and phosphate release induce the change to the closed form. Notably, specific mutations inhibit certain parts of this cycle in different ways. For example, although both K70A and T199A mutations of *E*DnaK inhibit ATP hydrolysis without disturbing ATP binding, only the former inhibits the structural change to the open form induced by ATP^12, 42, 43^. Here, we generated *T*DnaK mutants carrying the homologous mutations (K69A and T195A) and tested their properties. To remove the bound nucleotide, purified *T*DnaK and its mutants were incubated with 10 mM EDTA and subjected to size exclusion chromatography. The A_260_/A_280_ ratios of these proteins were approximately 0.6–0.7 (Fig. 1b,c). However, when these proteins were subjected to size exclusion chromatography followed by incubation with ATP, the ratios became approximately 1.0. The numbers of bound ATP per *T*DnaK were calculated from the difference spectra as 0.70, 0.89, and 0.64 for wild-type, K69A, and T195A, respectively, in this condition. Although wild-type *T*DnaK hydrolyzed ATP at 3.0 min^−1^ in the presence of saturating DnaJ and GrpE of *T. thermophilus* (*T*DnaJ and *T*GrpE), the rates of K69A and T195A were markedly lower, 0.32 and 0.38 min^−1^, respectively (Fig. 1d).

To determine the conformation of *T*DnaK, we constructed a fusion protein in which mseCFPΔC11 was inserted between Ala283 and Ser284 of *T*DnaK, a loop of the sub-domain II of NBD, cp173mVenus was fused after the Glu582 of *T*DnaK, and the *T*DnaK sequence followed by the Glu582 was removed (termed *T*K-FRET)^44–46^ (Fig. 1e). Through the conformational change, distance between these two fluorescent proteins would drastically change with the efficiency of fluorescence resonance energy transfer (FRET) expected to be high in the open conformation. We measured the emission spectrum of this protein with excitation at 435 nm, which excites the CFP fluorophore (Fig. 1f). Without nucleotide, the ratio of the fluorescence intensities of acceptor and donor (F_528_/F_475_) of *T*K-FRET was 1.4 (Fig. 1g). The ratio was increased to 1.7 after 5 min incubation with 3 mM ATP. Although the F_528_/F_475_ ratio (1.5) of *T*K-FRET carrying the K69A mutation in the *T*DnaK (*T*K_K69A-FRET) barely changed, the ratio (1.5) of *T*K_T195A-FRET was increased to 2.8 by adding 3 mM ATP (Fig. 1g). The ratio of the *T*K-FRET further increased to 2.2 by further addition of 1.0 μM *T*GrpE, while the ratios of the *T*K_K69A-FRET and the *T*K_T195A-FRET were unchanged. The time course of the F_528_ changes were also measured (Fig. 1h). By the addition of 3 mM ATP, the F_528_ of *T*K-FRET immediately increased 1.3-fold and subsequently decreased to near the original level within 5 min (Fig. 1h). Subsequent addition of *T*GrpE caused re-increase of the fluorescence intensity and the value was not so decreased. This was thought to reflect the ATP-induced opening, ATP-hydrolysis induced closing of SBD, and re-opening by the *T*GrpE-induced nucleotide exchange. Thus the intermediate increase of the F_528_/F_475_ ratio of *T*K-FRET observed in Fig. 1f,g would likely reflect a mixture of the open and closed form. Consistent with the results, the F_528_ of *T*K_K69A-FRET was unchanged by additions of ATP and *T*GrpE whereas that of *T*K_T195A-FRET increased approximately 1.4-fold by ATP addition and remained elevated, regardless of *T*GrpE. It should be noted that all the *T*K-FRETs were confirmed to be monomeric, though the ATP-induced dimerization of *E*DnaK was reported^47^ (Fig. S1). Thus, as expected, the K69A and T195A mutants of *T*DnaK appeared to have similar properties of the homologous K70A and T199A mutants of *E*DnaK.

### ATPase activity of ClpB is stimulated by covalently-fused DnaK variants

**Figure 2:**
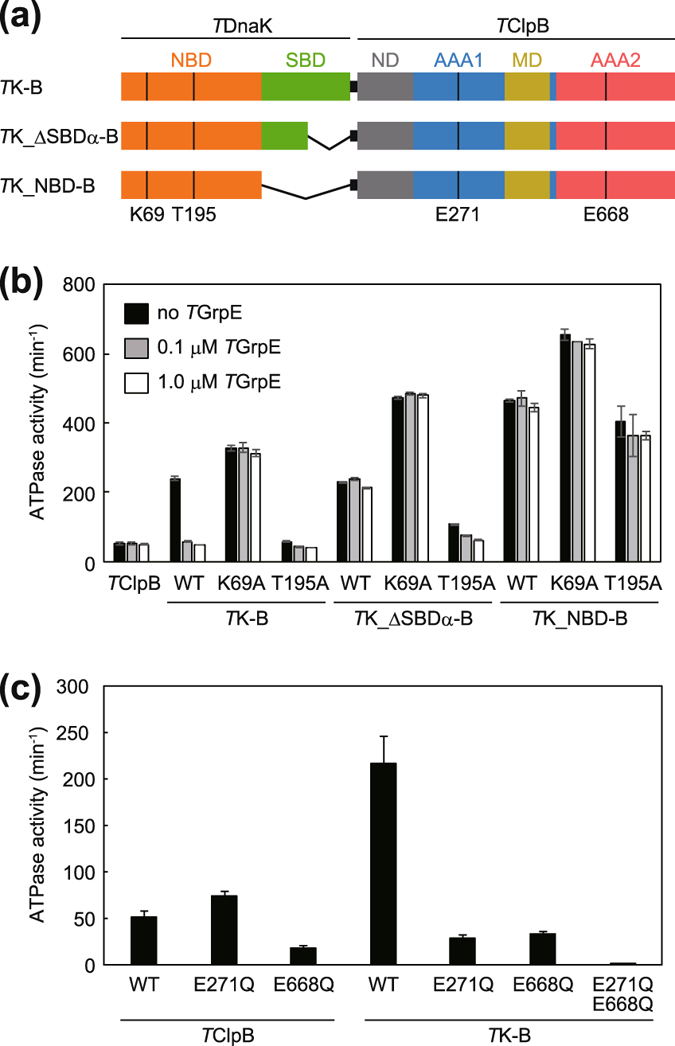
ATPase activities of fusion proteins. (**a**) Schematic drawing of the construction of *T*K-B, *T*K_ΔSBDα-B, and *T*K_NBD-B are shown. (**b**) ATPase activities of *T*K-B, *T*K_ΔSBDα-B, *T*K_NBD-B and their mutants carrying K69A and T195A mutations in the *T*DnaK portion. ATPase activities in the absence (black bar) or presence of *T*GrpE (0.1 μM; gray bar, 1.0 μM; open bar) are shown. (**c**) ATPase activities of *T*ClpB and *T*K-B and their mutants carrying E271Q and/or E668Q mutations in the *T*ClpB portion are shown. (**b**,**c**) The measurements were performed at 55 °C in the presence of 3 mM ATP by using an ATP regeneration system. Error bars represent standard deviations of three or more independent measurements. WT, wild-type.

Recently, it was found that DnaK had an ability to bind ClpB and stimulate its ATPase activity, although the binding affinity was low^41^. To clarify the state of DnaK able to perform this function, we constructed a series of fusion proteins in which *T*DnaK and *T*ClpB were connected by a 20-amino-acid-long flexible peptide linker (-ASGAGGSEGGGSEGGTSGAT-) that was used for the construction of tandemly fused ClpX^48^ (Fig. 2a). The length was 70 Å when fully extended and was sufficiently long that the NBD of *T*DnaK was able to interact with the MD of *T*ClpB.

At 55 °C, the wild-type *T*ClpB hydrolyzed ATP at 51 min^−1^ (Fig. 2b) whereas *T*DnaK showed no obvious ATPase activity; less than 0.1 min^−1^. When *T*DnaK was fused to the N-terminus of *T*ClpB, the fusion protein (termed *T*K-B) could hydrolyze ATP at a rate of about 5 times higher than that of *T*ClpB (Fig. 2b). Furthermore, when the K69A mutation of *T*DnaK was introduced into *T*K-B (termed *T*K_K69A-B), slightly higher ATPase activity was shown, indicating that the observed activity could mainly be attributed to the *T*ClpB portion. However, *T*K_T195A-B exhibited almost the same ATPase activity as that of *T*ClpB. Though, when 0.1 or 1.0 μM *T*GrpE was added, the ATPase activity of *T*K-B was decreased to that of unfused *T*ClpB, such a drastic decrease was not observed for *T*K_K69A-B (Fig. 2b). The absence of the ATPase stimulation of the *T*K_T195A-B or the *T*K-B with *T*GrpE intimated that the open form of *T*DnaK could not cause the stimulation. When the SBDα-truncated *T*DnaK was fused to *T*ClpB (termed *T*K_ΔSBDα-B), ATPase activity was stimulated as well as the case of *T*K-B, but the *T*GrpE-induced de-activation was not observed. *T*K_ΔSBDα-B having K69A mutation (termed *T*K_ΔSBDα_K69A-B) showed 1.5-fold higher ATPase activity than that of *T*K_K69A-B and *T*GrpE did not decrease the activity. The ATPase stimulation was also observed for the *T*K_ΔSBDα_T195A -B, though the extent was low, about two-fold. *T*GrpE slightly decreased the ATPase activity, but the effect was not so strict as observed for *T*K-B. Moreover, the ATPaes activity of the SBD-truncated version of the fusion protein (termed *T*K_NBD-B) was two-fold higher than that of the *T*K-B (Fig. 2b). In this case, neither the K69A nor the T195A mutations showed strict inhibition of the stimulation. The *T*GrpE hardly influence the ATPase activity of the *T*K_NBD-B and its mutants.

The mutations in the Walker B motif of the AAA1 (E271Q) and AAA2 (E668Q) of *T*ClpB caused ATPase defects of the corresponding AAA+ modules^23^. ATPase activities of these mutants were attributed to AAA2 and AAA1, respectively, and were not equivalently stimulated by *T*DnaK fusion (Fig. 2c). In addition, *T*K-B carrying both mutations (*T*K-B_E271Q_E668Q) showed no obvious ATPase activity, reconfirming that the ATPase activity of *T*K-B was mainly attributed to the *T*ClpB portion.

### Disaggregation activities of the fusion proteins

**Figure 3:**
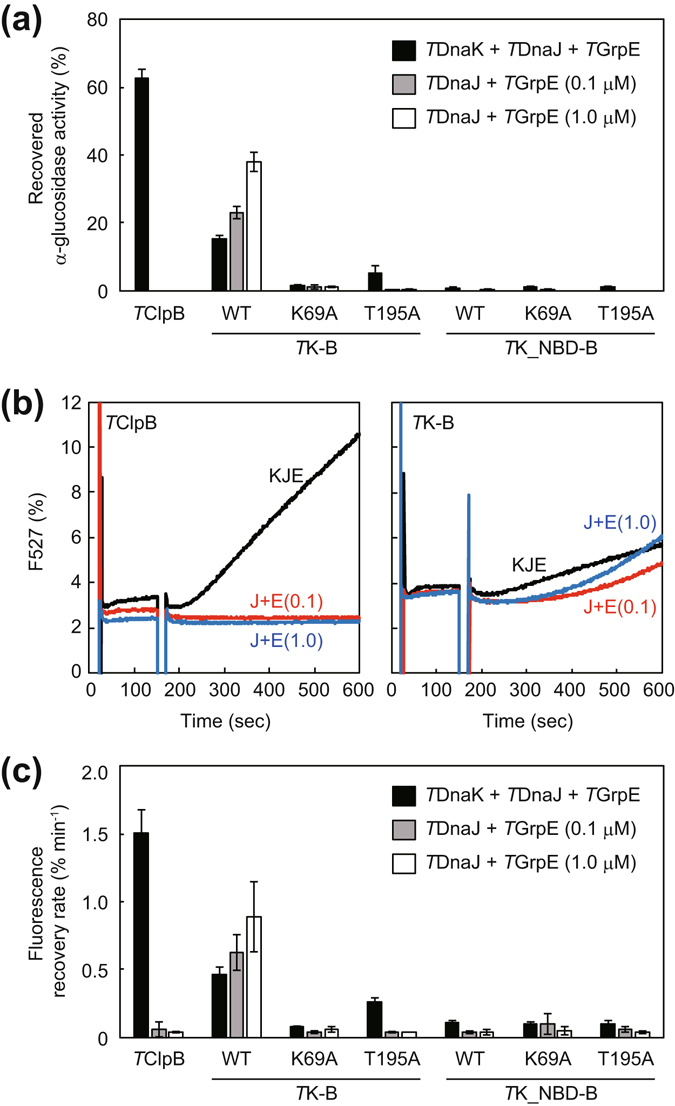
Disaggregation activities of fusion proteins. (**a**) Heat-aggregated α-glucosidase (0.2 μM) from moderate therrmophilic bacteria was incubated with *T*DnaK (0.6 μM), *T*DnaJ (0.2 μM), and *T*GrpE (0.1 μM dimer) (termed *T*KJE), and the indicated *T*ClpB or fusion proteins (0.05 μM hexamer) at 55 °C for 90 min in the presence of 5 mM ATP (black bar). After the incubation, the recovered enzymatic activity was measured and is shown as a percentage of the activity prior to heat aggregation. *T*DnaJ (0.2 μM) with *T*GrpE (0.1 μM dimer) (termed *T*JE0.1) (gray bar) or *T*DnaJ (0.2 μM) with *T*GrpE (1.0 μM dimer) (termed *T*JE1.0) (open bar) were also used instead of *T*KJE. (**b**) Heat-aggregated EYFP (0.3 μM) was incubated with chaperones at 55 °C in the presence of 5 mM ATP, and the fluorescence at 527 nm (excitation 513 nm) was monitored. *T*ClpB (left panel) and *T*K-B (right panel) were used. *T*KJE (black line), *T*JE0.1 (red line), or *T*JE1.0 (blue line) was also added. Fluorescence intensities are shown as a percentage of that prior to heat aggregation. (**c**) Initial rates (except for the lag times if existing) for the fluorescence recovery of EYFP were calculated from the measurement of (**b**) and the same experiments using *T*K-B, *T*K_NBD-B, and their mutants. Error bars represent standard deviations of three or more independent measurements. WT, wild-type.

α-Glucosidase of *Bacillus stearothermophilus* was completely aggregated by incubation at 73 °C for 10 min. If the aggregated protein was incubated with *T*DnaK, *T*DnaJ, *T*GrpE (termed *T*KJE) and *T*ClpB, at 55 °C for 90 min in the presence of 5 mM ATP, 63% of the protein was reactivated (Fig. 3a). When *T*K-B was used instead of *T*ClpB or *T*ClpB and *T*DnaK, 15% and 23% of the protein was reactivated, respectively. Thus, the fused *T*ClpB and *T*DnaK could cooperate properly whereas an additional unfused *T*DnaK appeared to injure the cooperation. Moreover, when the *T*GrpE concentration was increased to 1.0 μM, the reactivation yield was increased to approximately 38% in the absence of unfused *T*DnaK. *T*K_T195A-B showed a small disaggregation activity (approximately 5%) only in the presence of *T*KJE. All other fusion proteins showed no obvious disaggregation activity (less than 2%) in any conditions tested.

To investigate the disaggregation rate, we used EYFP as a substrate. Upon incubation at 80 °C for 15 min, EYFP was completely aggregated and lost its fluorescence. The recoveries of the fluorescence of the aggregated EYFP were monitored during the following co-incubation at 55 °C with chaperones and ATP, and the reactivation rates were calculated (Fig. 3b,c). When unfused *T*ClpB with *T*KJE were used, the EYFP fluorescence was recovered at 1.5% min^−1^ (the fluorescence of unheated EYFP was set as 100%) after an about 30-sec lag time. The EYFP reactivation rates by *T*K-B with *T*DnaJ and *T*GrpE in the presence or absence of unfused *T*DnaK were 0.46% and 0.63% min^−1^, respectively. As in the case of α–glucosidase, when the *T*GrpE concentration was high, *T*K-B could reactivate aggregated EYFP more effectively (0.88% min^−1^). It should be noted that the lag time for EYFP reactivation observed in the case of *T*K-B without unfused *T*DnaK was clearly longer than that of unfused chaperones. Specifically, the lag time was approximately 3 min and was slightly reduced with increased *T*GrpE concentration. Even in the case of EYFP, *T*K_T195A-B showed a small disaggregation activity (approximately 0.26% min^−1^) only in the presence of *T*KJE. All other fusion proteins showed no obvious disaggregation activity (approximately 0.1% min^−1^ or less) in any conditions.

### Characterization of the repressed and the hyperactive mutants of *T*ClpB

**Figure 4:**
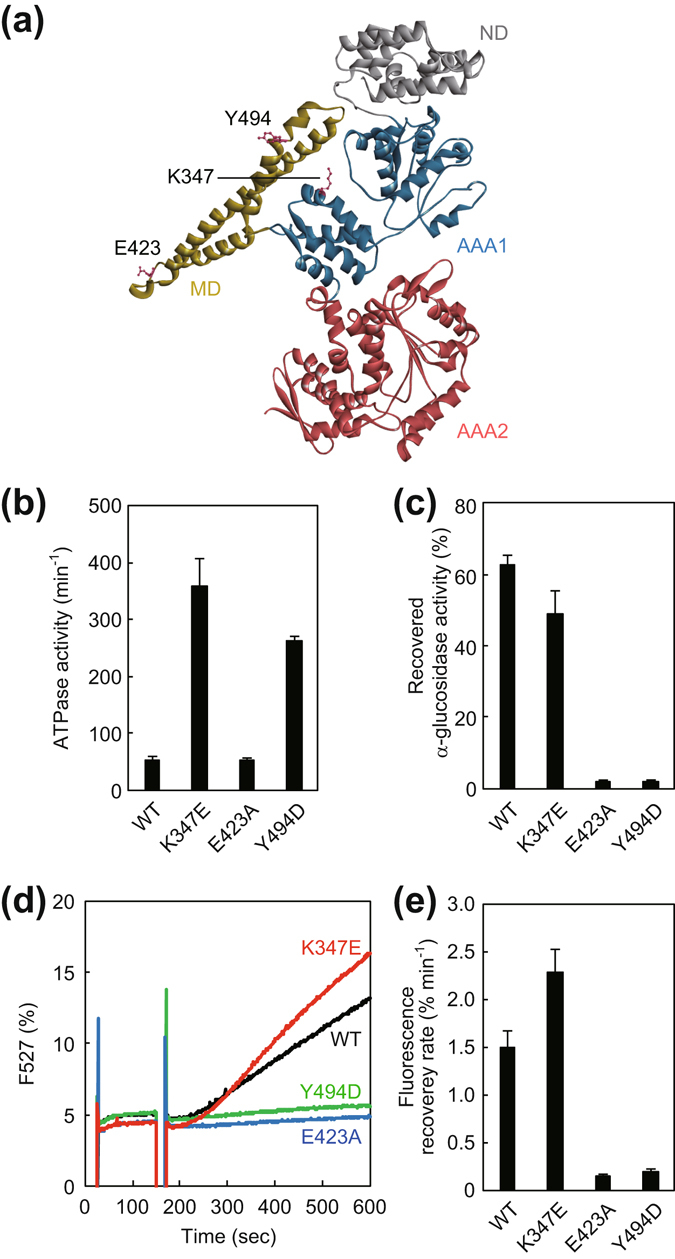
Characterization of hyperactive and repressed mutants of *T*ClpB. (**a**) Monomeric structure of *T*ClpB (PDBid:1QVR)^21^ is shown. The N-domain (ND), M-domain (MD), AAA1, and AAA2 are colored by gray, yellow, blue, and red, respectively. The mutated residues, Lys347, Glu423, and Tyr494 are shown as sticks. (**b**) ATPase activities of the *T*ClpB mutants were measured. The experimental procedure was the same as in Fig. 2. (**c**) Disaggregation activities of the *T*ClpB mutants with *T*DnaK (0.6 μM), *T*DnaJ (0.2 μM), and *T*GrpE (0.1 μM dimer) (termed *T*KJE) were measured by using α-glucosidase as a substrate. The experimental procedure was the same as in Fig. 3a. The time courses (**d**) and the initial rates (**e**) of the reactivation of heat-aggregated EYFP by the *T*ClpB mutants with *T*KJE are shown. The experimental procedure was the same as in Fig. 3b,c. Error bars represent standard deviations of three or more independent measurements. WT, wild-type.

E432A mutation of *E*ClpB has been shown to cause loss of its disaggregation activity and was thought to strengthen inter-subunit MD-MD interaction and stabilize horizontal conformation^28, 39^. Conversely, R356E and Y503D mutants of *E*ClpB showed vastly enhanced ATPase activities^38, 40^. Although the R356E mutant exhibited high chaperone activity, the Y503D mutant lost the ability to bind DnaK because Y503 constitutes a part of the DnaK binding site^41^, and therefore lost chaperone activity as well. Here, we prepared homologous *T*ClpB mutants, E423A, K347E, and Y494D, respectively, and tested their properties (Fig. 4). Although the E423A mutant showed almost the same ATPase activity as that of wild-type, those of K347E and Y494D mutants were 5 to 8-times higher (Fig. 4b). The E423A and Y494D mutants were unable to reactivate either the aggregated α-glucosidase or EYFP, whereas the K347E mutant could significantly reactivate both, in the presence of ATP and *T*KJE (Fig. 4c,d,e). Specifically, the reactivation yield of α-glucosidase by the K347E mutant was 80% that of the wild-type, whereas the EYFP reactivation rate was 1.5 times higher than wild type levels. When the aggregated EYFP was incubated with *T*ClpB mutants in the absence of *T*KJE, small reactivations were observed in the case of K347E (0.57% min^−1^) and Y494D (0.25% min^−1^) but the reactivation rates of wild type and E423A were less than 0.1% min^−1^. These results were consistent with previous reports of *E*ClpB^38–40^.

### Activated ClpB can reactivate the fused EYFP aggregation

**Figure 5:**
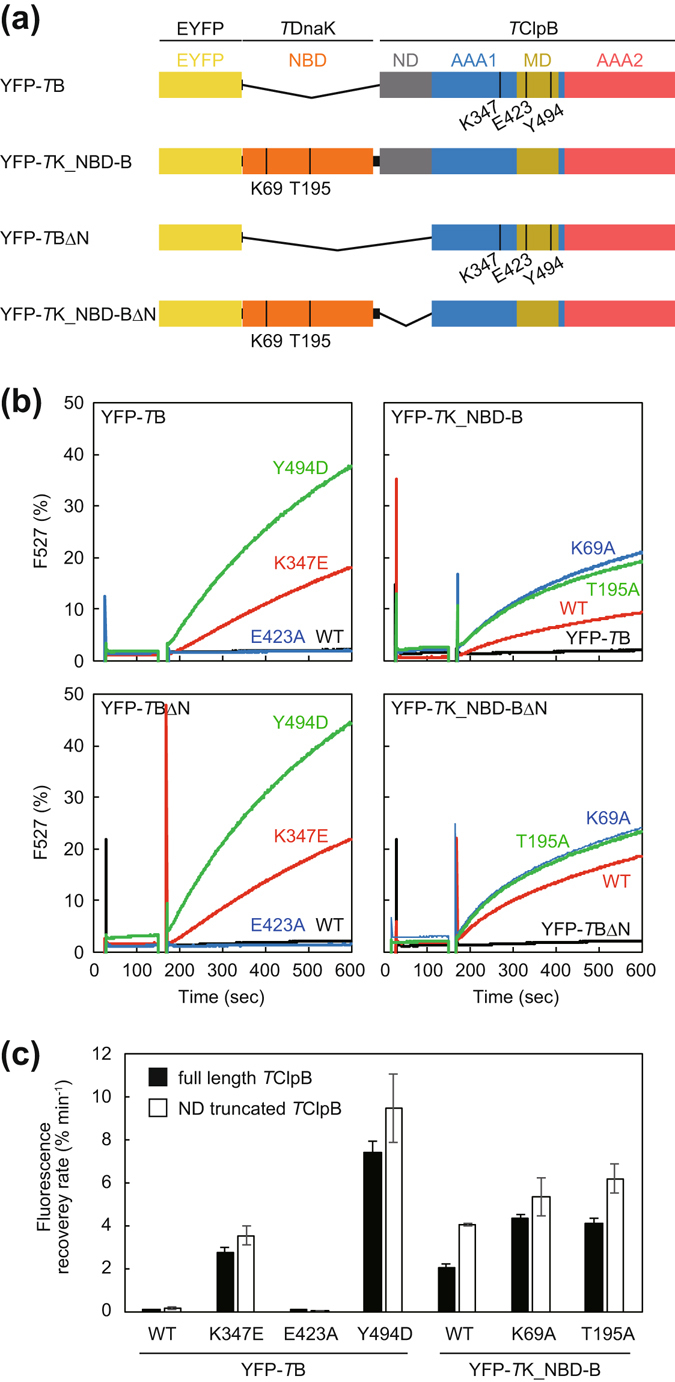
Disaggregation reaction of aggregated EYFP tethered by a chaperone. (**a**) Schematic drawing of the construction of YFP-*T*B and YFP-*T*K_NBD-B are shown. (**b**) YFP-*T*B (left panel), YFP-*T*K_NBD-B (right panel), and their mutants (6.0  μM monomer) were heat treated at 80 °C for 15 min and the EYFP portions of these proteins were aggregated. The heat-treated fusion proteins were diluted to 0.3 μM monomer, incubated at 55 °C, and the fluorescence at 527 nm (excitation 513 nm) was monitored. After 2 min incubation, ATP (final concentration was 5 mM) was added and the fluorescence monitoring was continued. Fluorescence intensities are shown as a percentage of that prior to heat aggregation. (**c**) Initial rates of the recovery of the fluorescence were calculated from the measurements shown in (**b**). Error bars represent standard deviations of three or more independent measurements. WT, wild-type.

It has been shown that the ATPase activity of ClpB is stimulated by adding a large amount of DnaK^41^. However, it is not clear whether the ATPase activation correlates to the disaggregation reaction or whether the hyperactive mutants of ClpB strictly reproduce the activated state induced by DnaK binding. To answer these questions, we prepared fusion proteins in which EYFP was fused to the N-terminus of *T*K_NBD-B, *T*ClpB, and its repressed/hyperactive mutants (Fig. 5a). As the chaperones were derived from thermophilic bacteria, only the EYFP portions were aggregated by the incubation at 80 °C for 15 min (Fig. S2). When the EYFP was fused to wild-type or the repressed mutant of *T*ClpB, the fluorescence of the aggregated EYFP was not recovered by the subsequent incubation at 55 °C with ATP (Fig. 5b,c). However, the hyperactive mutants YFP-*T*ClpB_K347E and YFP-*T*ClpB_Y494D could reactivate the fused aggregated EYFP at 2.8- and 7.3-% min^−1^, respectively. YFP-*T*K_NBD-B, YFP-*T*K_NBD_K69A-B, and YFP-*T*K_NBD_T195A-B could also reactivate the fused aggregation, at 1.9-, 4.4-, and 4.0-% min^−1^. It should be noted that the lag phase seen in the time courses of unfused-EYFP disaggregation was not observed in all the time courses of the fused EYFP (Fig. 5b). We also prepared an ND-truncated version of EYFP fused proteins and measured the disaggregation rates of the fused EYFP as above (Fig. 5b,c). The rates of all the ND-truncated EYFP fusion proteins were more rapid than those of the corresponding fusion proteins containing the ND. Moreover, the statistical analysis demonstrated that the differences between YFP-*T*K_NBD-B and YFP-*T*K_NBD-B_ΔN, or YFP-*T*K_NBD_T195A-B and YFP-*T*K_NBD_T195A-B_ΔN were significant (P < 0.01).

It should be noted that we confirmed all the fusion proteins used here formed hexameric structure or close to it (Fig. S3).

## Discussion

Rosenzweig *et al*. showed direct interaction between DnaK and ClpB by NMR study, and demonstrated that DnaK stimulated the ATPase activity of ClpB^41^. However, the stimulation is observed only in the high protein concentrations, as the affinity of ClpB for DnaK is low. Here, we constructed a fusion protein consisting of *T*DnaK and *T*ClpB, and found that the ATPase activity of the *T*ClpB portion was perpetually activated. This fusion protein therefore appeared to represent a good tool to clarify the details of the activation and its contribution to the disaggregation reaction.

**Figure 6:**
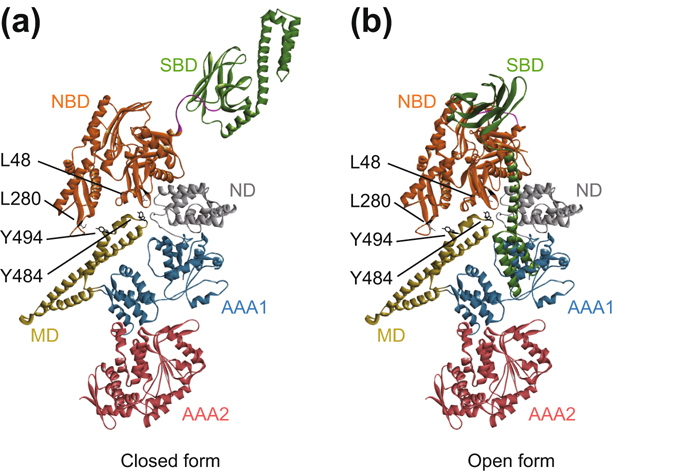
Models for the docked structure of *T*DnaK and *T*ClpB. Structural models of the closed (**a**) and the open (**b**) form of *T*DnaK were arrayed on the *T*ClpB structure as seen in the binding model proposed by NMR study^41^.

The ATPase activation of *T*K-B was severely inhibited by the *T*GrpE addition or the T195A mutation. In contrast, *T*K_K69A-B showed the stimulation that was not inhibited by *T*GrpE. These results suggested that DnaK in the open form could not activate ClpB. In the case of *T*K_ΔSBDα-B, the *T*GrpE-induced de-activation was disappeared, and the T195A mutant showed ATPase stimulation though the extent was low. Moreover, all the *T*K_NBD-B and its mutants showed highly stimulated ATPase activity, and these activities were hardly decreased even in the presence of 1.0 µM *T*GrpE. These results indicated that the SBD, especially the SBDα in the open form, inhibited the DnaK-ClpB interaction. Previous NMR study indicated that the L48 and L280 of *T*DnaK interact with the Y484 and Y494 of *T*ClpB, respectively^41^. If the structural models of the open and closed form of *T*DnaK were each arrayed to the structural model of *T*ClpB such that the interacting residues were close to each other, the SBDα of DnaK and AAA1 of ClpB would cause a steric crash only in the case of the open form (Fig. 6). This was in good agreement with our results.

In this model, the distance between Ala595 of *T*DnaK and Glu4 of *T*ClpB were about 26 Å and 98 Å for the open and the closed form, respectively. The total length of the C-terminal flexible region (Asn596-Asp615) of *T*DnaK, the 20-amino-acid linker between *T*DnaK and *T*ClpB, and the N-terminal flexible region (Met1-Asn3) of *T*ClpB were about 156 Å. Taking into account the flexibility of the relative positions of SBD and ND, the length of the linker would be enough long to cause the conformational change of *T*DnaK and represent the structures of the models in Fig. 6. This reconfirmed the adequacy of the linker used here.

The *T*K-B mutants carrying an ATPase-defective mutation in the AAA1 or the AAA2 of the *T*ClpB portion showed no obvious ATPase activation, suggesting that DnaK influenced the inter-domain communication between these two AAA+ modules to activate ClpB. This is in good agreement with the recent observation that some MD mutations influence the inter-domain communication between these two AAA+ modules^49^.

In the presence of *T*KJE, *T*K-B showed a recognizable disaggregation activity, which was slightly increased when the unfused *T*DnaK was omitted. These results indicated that the fused *T*DnaK and *T*ClpB can cooperate properly. The inhibitory effect of the additional unfused *T*DnaK is likely caused by the competition with fused *T*DnaK, which preferentially interacts with the fused *T*ClpB for aggregation binding. The *T*K_T195A-B showed a small disaggregation activity only in the presence of *T*KJE. The *T*DnaK portion preferred the open conformation and its NBD would not interact with the MD. Thus, the unfused *T*DnaK would be able to cooperate with the *T*ClpB portion, although the efficiency was low. This assumption was consistent with that the *T*K_T195A-B showed no disaggregation activity in the absence of unfused *T*DnaK, and that *T*K_K69A-B, *T*K_NBD-B, and its mutants with *T*DnaJ and *T*GrpE showed no obvious activity regardless of the presence of unfused *T*DnaK. These results also indicated that the activation of ClpB is not sufficient to execute the disaggregation process. Taken together, our findings demonstrate that for effective disaggregation, ClpB should interact with an intact DnaK that can bind aggregation efficiently.

On the EYFP disaggregation by *T*ClpB with *T*KJE, an approximately 30-sec lag time between the addition of chaperones and the fluorescence recovery initiation was observed. Such a lag time was also observed for the GFP disaggregation by *E*ClpB with *E*DnaK and its co-factors^50^. The lag time disappeared following the pre-incubation of the GFP aggregation with *E*DnaK and co-factors, but not with *E*ClpB, demonstrating that DnaK was responsible for an event in the early stage of disaggregation. On the disaggregation by *T*K-B with *T*DnaJ and *T*GrpE, the lag time was prolonged, indicating that the fused *T*DnaK reduced the ability to perform the event and that the event itself might consist of the binding and/or holding of an aggregation. For *T*K-B, the ATPase cycle of the *T*DnaK portion would become non-optimal to bind and hold an aggregation, as the binding sites of DnaK for ClpB and GrpE were mostly overlapped^41, 51^ and the access of *T*GrpE to the *T*DnaK would be reduced by the fused *T*ClpB that would not accelerate the nucleotide exchange of DnaK^41^. This was in good agreement with the observation that the addition of excess amounts of *T*GrpE could shorten the lag time albeit partially. From the point of view of disaggregation efficiency, the optimum *T*GrpE concentration (more than 1.0 μM) for the *T*K-B was also much greater than that for the unfused chaperones (approximately 0.1 μM)^52^. In contrast, the ATPase activation observed for *T*K-B was abrogated by adding comparable concentrations of *T*GrpE. It therefore appears that the proper proceeding of the ATPase cycle of DnaK, especially in the early stage of disaggregation, rather than the continuous activation of ClpB, is important for effective disaggregation.

Although the hyperactive mutants of ClpB were previously thought to mimic the DnaK-induced activated state, the data supporting this assumption were insufficient. Here, we constructed a repressed (E423A) and two hyperactive (K347E and Y494D) mutants of *T*ClpB and confirmed that they showed similar properties of the corresponding *E*ClpB mutants^38–40^. The ATPase activities of these two hyperactive mutants were 5 to 8 times higher than that of wild-type and similar to those of *T*K-B and *T*K_NBD-B. The lack of chaperone activity of *T*ClpB_Y494D was explained by the mutated residue being responsible for the interaction with DnaK^41^. The lack of chaperone activity of *T*K-NBD-B and its mutants may also be explained by the inhibition of intact, unfused *T*DnaK binding to the *T*ClpB portion, as the fused NBD constantly occupied the MD. This was also consistent with a recent report in which the de-repression of ClpB by itself could not bypass the DnaK requirement for disaggregation^53^.

By fusing EYFP, we successfully placed the aggregation on the N-terminal side of *T*ClpB. Although the wild-type and the repressed mutant could not reactivate the tethered aggregation, all the ATPase-activated *T*ClpBs including the hyperactive mutants, *T*K_NBD_B, and its mutants could effectively disaggregate them without *T*KJE. These results indicated that the activated ClpB can perform disaggregation reactions, if the aggregation is placed very close to the chaperone. These results also strongly supported the assumption that the hyperactive mutants mimic the ClpB activated by DnaK.

In the case of disaggregation of the chaperone-fused EYFP, a lag time was not observed and the overall disaggregation rates were appreciably higher than those mediated by the EYFP-unfused chaperones. It should be noted that a 30-sec lag time was observed even in the case of the unfused K347E hyperactive *T*ClpB with *T*DnaK. These results were consistent with the above assumption that the length of the lag time were influenced by the ability of DnaK to bind and hold aggregations. Consequently, the importance of the appropriate rate of the ATPase cycle (i.e., conformational change) of DnaK in the early stage of disaggregation, was reconfirmed. Furthermore, the prior proposal that the GrpE was only needed for the reactivation of substrate proteins after disaggregation was based solely on experiments using luciferase as substrate^41^, whereas our results suggest that the GrpE is required in the early stage of disaggregation. The requirement of GrpE might therefore differ according to substrates and denaturing conditions.

Overall, the disaggregation rates of hyperactive YFP-*T*Bs are faster than those of YFP-*T*K_NBD-Bs. In the YFP-*T*K_NBD-Bs, the interaction between aggregated EYFP and *T*ClpB might be weakened by the insertion of a 41-kDa NBD. Moreover, in all EYFP-fused chaperones, the disaggregation rates increased when the ND of the *T*ClpB portion was deleted. As the ND was shown to support substrate binding of ClpB but not to be essential for the disaggregation activity^27, 32–36^, it therefore likely just functions as a spacer between the aggregation and the AAA1 pore in a condition where the aggregation was already tethered to ClpB. Additionally, the distance between the aggregation and the AAA1 pore of ClpB may constitute important factor, even though all rate-determining factors are not yet elucidated.

**Figure 7:**
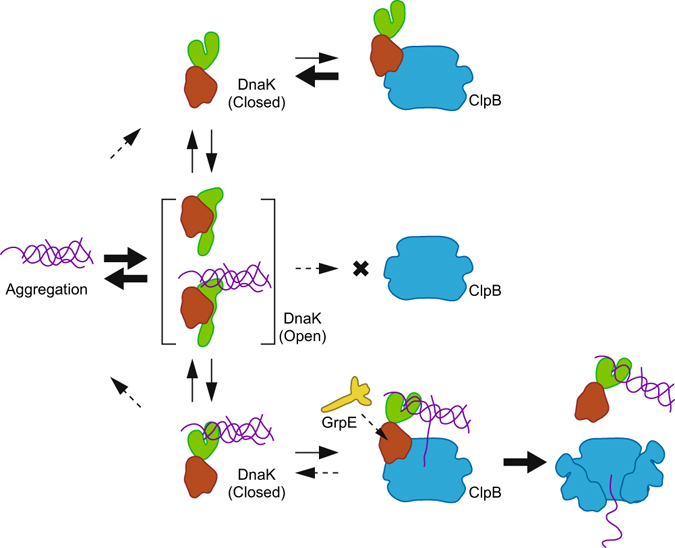
Models for the interactions between chaperones and protein aggregations. DnaK cycles conformational changes between open and closed forms. Although the open form can readily bind and release aggregated proteins, the closed form does not. The closed form of DnaK can bind ClpB and activate it; however, owing to the low affinity, the complex readily dissociates. However, if the bound DnaK holds an aggregation, the ClpB can start threading it. During the threading, the DnaK is tethered by the aggregation to the ClpB and the interaction between NBD and MD of these chaperones is maintained. Consequently, the NBD-MD interaction reduces the approach of GrpE to the DnaK molecule; therefore, the DnaK would maintain the closed form. Thus, the trigonal complex consisting of DnaK, ClpB, and protein aggregation is stabilized. The dissociation of this complex would be effected by accomplishment of the threading and/or collapse of the ClpB ring.

The model of functional interactions between chaperones during the disaggregation process revealed by this study is shown in Fig. 7 i) DnaK cycles ATP-driven conformational changes under the control of co-factors and binds protein aggregations. ii) The NBD of the ADP-bound closed form of DnaK interacts with the MD of ClpB and converts it to an activated state. iii) If the DnaK had already bound to an aggregation, the activated ClpB starts threading the aggregation through its central pore. iv) During the threading process, the aggregation would not be released by the closed form of DnaK; thus, the NBD-MD interaction would be stabilized by the tethering effect mediated by the aggregation. v) Simultaneously, the NBD-MD interaction might reduce the approach of GrpE to the DnaK molecule; therefore, DnaK would maintain the closed form, such that the aggregation is stably held. vi) Through accomplishment of the threading and/or collapse of the ClpB ring, the DnaK would be released from the ClpB. Previously, it was reported that the ClpB ring was fragile and easily dissociated to avoid jamming by the extremely stable aggregation^54–56^. In the step iii)–v), ClpB would thread a polypeptide other than the polypeptide bound to the DnaK, and might cause the peeling of the aggregation. Alternatively, by pulling the polypeptide bound to the DnaK, ClpB might stretch the shrinking polypeptide in the aggregation.

In our model, only DnaK in the closed form can interact and activate ClpB, and only DnaK grabbing the aggregation can maintain the interaction with ClpB. These properties enable the chaperones to proceed with timely activation and prevent wasteful consumption of ATP. Our data of EYFP-fused chaperones demonstrated that all the required roles of DnaK during the disaggregation reaction could be accomplished by simply placing the aggregation near the ClpB along with simultaneous activation of the ClpB. The mutually regulated interactions of the chaperones proposed in the model enable DnaK to fulfill its roles precisely.

## Methods

### Plasmids

A DNA fragment containing the *T*DnaK gene carrying mutations that removed two internal restriction sites, one of three SacII sites, and one of two AatII sites without changing the amino acid sequence was prepared by overlap extension PCR^57, 58^. The plasmid pMDK6^59^ was used as a PCR template. The resulting DNA fragment was ligated into the NdeI and EcoRI sites of pET23a to construct the plasmid pET23a-*T*DnaK. A DNA fragment containing the gene of the fusion protein *T*K-FRET in which 6 histidines, Met1-Ala283 of *T*DnaK, 3 glycines, mseCFPΔC11, 3 glycines, Ser284-Glu582 of *T*DnaK, 3 glycines, and cp173mVenus were fused in the given order was amplified by overlap extension PCR. The plasmids pET23a-*T*DnaK and pRSET_A team1.03^46^ were used as PCR templates. The fragment was ligated into NdeI and BamHI sites of pET23a to construct the expression vector pET23a-*T*K-FRET. DNA fragments containing the genes of *T*K-B, *T*K_ΔSBDα-B and *T*K_NBD-B, in which *T*DnaK or ΔSBDα (Met1-Leu501) or NBD (Met1-Met377) of *T*DnaK were fused to *T*ClpB by using a peptide linker, -ASGAGGSEGGGSEGGTSGAT-, were also amplified. The plasmids pMDK6, pMCB1^4^, and pIDTSMART-AMP_TKApaI_L_TBdNNsiI_BamHI containing the chemically synthesized gene of the peptide linker were used as PCR templates. The fragments were ligated into the NdeI and EcoRI sites of pET23a and pET21c, respectively to construct the expression vectors pET23a-*T*K-B, pET23a-*T*K_ΔSBDα-B and pET21c-*T*K_NBD-B. A DNA fragment of the EYFP gene (a GFP mutant carrying S65G/V68L/S72A/T203Y substitutions) was prepared by overlap extension PCR and ligated into the NdeI and EcoRI sites of pET23a to construct the expression plasmid pET23a-EYFP. A DNA fragment containing the gene of YFP-*T*K_NBD-B in which EYFP, 3 glycines, NBD of *T*DnaK, a peptide linker, and *T*ClpB were arrayed in the given order was amplified by overlap extension PCR. A DNA fragment containing the gene of YFP-*T*B that consists of EYFP, 3 glycines, and *T*ClpB was also amplified. The plasmids pET23a-EYFP, pMDK6, pMCB1, and pIDTSMART-AMP_TKApaI_L_TBdNNsiI_BamHI were used as PCR templates. These DNA fragments were ligated into the NdeI and EcoRI sites of pET21c and pET23a, respectively to construct the expression vectors pET21c-EYFP-*T*K_NBD-B and pET23a-EYFP-*T*B. Site-directed mutagenesis was performed by the overlap extension PCR method. The DNA sequences of these expression plasmids were confirmed by DNA sequence analysis.

### Proteins

Rabbit pyruvate kinase and chicken lactate dehydrogenase were purchased from Oriental Yeast. α-glucosidase from *B. stearothermophilus* was purchased from Sigma. *T*DnaK, *T*DnaJ, *T*GrpE, *T*ClpB, and their mutants were expressed in *E. coli* BL21(DE3) or KRX carrying pMDK6, pMDJ10^59^, pMGE3^60^, or pMCB1, and purified as described previously^25, 61, 62^.

*T*K-FRET and its mutants were expressed in *E. coli* BL21(DE3). The cells were suspended in 25 mM Tris-HCl (pH 7.5) and disrupted by sonication. The cell extracts were centrifuged at 9,100 *g* for 30 min at 4 °C. The supernatant was filtered using a 0.22-µm PES Syringe Filter (Starlab Scientific) and was applied to a HiTrap^TM^ DEAE FF column (GE Healthcare) equilibrated with 25 mM Tris-HCl (pH 7.5). The column was washed with the same buffer and eluted with the same buffer containing 1 M NaCl. Fractions containing *T*K-FRETs were applied to HisTrap^TM^ HP (GE Healthcare) equilibrated with 25 mM Tris-HCl (pH 7.5), 300 mM NaCl, and 10 mM imidazole. The column was washed with the same buffer and eluted with the same buffer containing 250 mM imidazole. The eluted *T*K-FRETs were concentrated to 5–10 mg·ml^−1^ by using a 30 K Amicon ultra device (Merck Millipore).

To remove the bound nucleotides, *T*DnaK, *T*K-FRET, and their mutants were incubated with 10 mM EDTA at 55 °C for 60 min and then centrifuged at 21,000 *g* for 3 min. The solution was concentrated to 15–30 mg·ml^−1^ by using a 30 K Amicon ultra device and was centrifuged at 21,000 *g* for 1 min. An aliquot (250 μl) was loaded on the HPLC gel filtration column Superdex^TM^ 200 10/300 GL (GE Healthcare) equilibrated with 25 mM Tris-HCl (pH 7.5), 150 mM KCl, and 10 mM EDTA at room temperature. The fractions containing the proteins were applied to an NAP5 gel-filtration column (GE Healthcare) equilibrated with 50 mM 3-N-morpholinopropanesulfonic acid (MOPS)-NaOH (pH 7.5), 150 mM KCl, and 5 mM MgCl_2_. The fractions containing proteins were frozen with liquid nitrogen and stored at −80 °C until use.

*T*K-B, *T*K_ΔSBDα-B, *T*K_NBD-B, YFP-*T*B, YFP-*T*K_NBD-B, and their mutants were expressed in *E. coli* BL21(DE3) or KRX. The cells were suspended in 25 mM Tris-HCl (pH 7.5), 1 mM EDTA and disrupted by sonication. The cell extracts were incubated at 80 °C (for *T*K-B, *T*K_ΔSBDα-B, *T*K_NBD-B, and their mutants) or 60 °C (for YFP-*T*B, YFP-*T*K_NBD-B and their mutants) for 30 min, and centrifuged at 9,100 *g* for 30 min at 4 °C. The supernatant was filtered using a 0.22-µm PES Syringe Filter and was applied to a DEAE Toyopearl column (Tosoh) equilibrated with 25 mM Tris-HCl (pH 7.5) and 1 mM EDTA. The column was washed with the same buffer and eluted with the same buffer containing 300 mM NaCl. Fractions containing the proteins were pooled, and solid ammonium sulfate was added to a concentration of 400 mM. In the case of *T*K-B, the final concentration of ammonium sulfate was 600 mM. These solutions were applied to a Butyl Toyopearl column (Tosoh) equilibrated with 25 mM Tris-HCl (pH 7.5), 1 mM EDTA, 400- or 600-mM ammonium sulfate, and eluted with a linear reverse gradient of ammonium sulfate (400–0 mM). In the case of *T*K-B_E271Q and *T*K-B_E668Q, further purification was performed with a Superdex^TM^ 200 10/300 GL HPLC gel filtration column equilibrated with 50 mM MOPS-NaOH (pH 7.5), 500 mM KCl, 5 mM MgCl_2_, and 2 mM ADP. In the case of YFP-*T*K_NBD-B, further purification was performed with a Superose 6 HPLC gel filtration column (GE Healthcare) equilibrated with 50 mM MOPS-NaOH (pH 7.5), 150 mM KCl, 5 mM MgCl_2_, and 2 mM ATP. The purified proteins were applied to a PD10 gel-filtration column (GE Healthcare) equilibrated with 50 mM MOPS-NaOH (pH 7.5), 150 mM KCl, and 5 mM MgCl_2_. The fractions containing proteins were frozen with liquid nitrogen and stored at −80 °C until use.

EYFP was expressed in *E. coli* BL21(DE3). The cells were suspended in 25 mM Tris-HCl (pH 7.5), 1 mM EDTA, and 1 mM DTT and disrupted by sonication. The cell extract was incubated at 65 °C for 20 min and centrifuged at 15,000 *g* for 30 min at 4 °C. The supernatant was filtered using a 0.22-µm PES Syringe Filter and applied to a DEAE Toyopearl column equilibrated with 25 mM Tris-HCl (pH 7.5), 1 mM EDTA, and 1 mM DTT. The column was washed with the same buffer containing 20 mM NaCl and eluted with the same buffer containing 60 mM NaCl. Fractions containing EYFP were pooled and solid ammonium sulfate was added to a concentration of 800 mM. The solution was applied to a Butyl Toyopearl column equilibrated with 25 mM Tris-HCl (pH 7.5), 1 mM EDTA, 1 mM DTT, and 800 mM ammonium sulfate, and eluted with a linear reverse gradient of ammonium sulfate (800–0 mM). The purified EYFP was applied to a PD10 gel-filtration column equilibrated with 50 mM MOPS-NaOH (pH 7.5), 150 mM KCl, and 5 mM MgCl_2_. The fractions containing EYFP were frozen with liquid nitrogen and stored at −80 °C until use.

### Measurement of ATP binding

*T*DnaK and its mutants (10 mg·ml^−1^) were incubated with 3 mM ATP at 55 °C for 60 min. The mixture was centrifuged for 1 min at 21,000 *g*. An aliquot (100 μl) was loaded on the HPLC gel filtration column TSK G-3000SWXL (Tosoh) equilibrated with 50 mM MOPS-NaOH (pH 7.5), 150 mM KCl, and 5 mM MgCl_2_ at room temperature. The eluted *T*DnaKs were concentrated to approximately 1.7 mg·ml^−1^ by using a 30 K Amicon ultra device. The absorption spectra of these solutions were measured using a V-650 spectrophotometer (JASCO). From the difference spectrum between the spectrum of the same proteins with or without ATP incubation, the amounts of bound ATP were calculated using the extinction coefficient of ATP (ε_259 nm_ = 15,400 M^−1^ cm^−1^).

### ATPase activity

ATPase activities of chaperones were measured spectrophotometrically in an ATP-regenerating system at 55 °C. The reaction mixture consisted of 50 mM MOPS-NaOH (pH 7.5), 150 mM KCl, 5 mM MgCl_2_, 2.5 mM phosphoenolpyruvate, 0.2 mM NADH, 50 µg·ml^−1^ pyruvate kinase, 50 µg·ml^−1^ lactate dehydrogenase, and 3 mM ATP. Chaperones were added to the reaction mixture and the changes in absorbance at 340 nm were monitored using a V-650 spectrophotometer.

### Measurements of FRET

*T*K-FRET (0.6 μM) and its mutants dissolved in 50 mM MOPS-NaOH (pH 7.5), 150 mM KCl, and 5 mM MgCl_2_ were incubated at 55 °C for 3 min. Subsequently, 3 mM ATP were added and incubated for 5 min. Furthermore, *T*GrpE (final concentration 1.0 μM) was added and incubated for 2 min. After each incubation, the fluorescence spectra were measured. Excitation wavelength was 435 nm. The FRET efficiencies were expressed by the ratio of fluorescence intensities at 528 nm and 475 nm. *T*K-FRET (0.6 μM) and its mutants dissolved in 50 mM MOPS-NaOH (pH 7.5), 150 mM KCl, and 5 mM MgCl_2_ were incubated at 55 °C for 3 min. Subsequently, the monitoring of the fluorescence intensity was initiated. After 60-sec monitoring, 3 mM ATP was added and the monitoring was continued for 200 s at 55 °C. After another 30 s, *T*GrpE (final concentration 1.0 μM) was added and the monitoring was continued for 300 s at 55 °C. The excitation and emission wavelengths were 435 nm and 528 nm, respectively. Fluorescence measurements were performed using an FP-8500 spectrofluorometer (JASCO).

### α-Glucosidase disaggregation

α-Glucosidase (0.2 μM monomer) in a mixture containing 50 mM MOPS-NaOH (pH 7.5), 150 mM KCl, 10 mM MgCl_2_, 5 mM ATP, and 5 mM tris-(2-carboxyethyl) phosphine hydrochloride (TCEP) were heat aggregated by incubation at 73 °C for 10 min. Subsequently, the indicated chaperones were added to the reaction mixture and the mixture was incubated at 55 °C for 90 min. Recovered enzymatic activity was assayed as described previously^34^. The recovered activities were expressed as percentages of the enzymatic activity prior to heat-aggregation.

### EYFP disaggregation

EYFP (6.0 μM monomer) in a mixture containing 50 mM MOPS-NaOH (pH 7.5), 150 mM KCl, 10 mM MgCl_2_, and 5 mM TCEP was heat aggregated by incubation at 80 °C for 15 min. The aggregated EYFP was diluted 20-fold into the same buffer containing 5 mM ATP and the monitoring of the EYFP fluorescence was initiated. After 2-min incubation at 55 °C, indicated chaperones were added to the mixture and the incubation was continued. In the case of chaperone-fused EYFP, the dilution buffer did not contain ATP, and 5 mM ATP was added after the 2-min incubation at 55 °C. The excitation and emission wavelengths were 513 nm and 527 nm, respectively. Fluorescence measurements were performed using an FP-6500 spectrofluorometer (JASCO). To exclude the effects of lag time, the disaggregation rates were estimated as follows. Slopes of a fluorescence change were calculated by using any consecutive 1-min data in the initial 7-min measurement after starting the reaction. The slope showing the maximum value in a measurement was used as the rate of the measurement.

## Electronic supplementary material

Fusion protein analysis reveals the precise regulation between Hsp70 and Hsp100 during protein disaggregation
