# Supplementary material for: Fusion protein analysis reveals the precise regulation between Hsp70 and Hsp100 during protein disaggregation
Source: Sci Rep. 2017 Aug 17;7:8648. doi: 10.1038/s41598-017-08917-8 (PMC5561102; doi:10.1038/s41598-017-08917-8)
Supplement: Supplementary file 1 — Fusion protein analysis reveals the precise regulation between Hsp70 and Hsp100 during protein disaggregation [file 41598_2017_8917_MOESM1_ESM.pdf]

Supplementary Information

Fusion protein analysis reveals the precise regulation between Hsp70 and Hsp100 during protein disaggregation

Sayaka Hayashi[1,4], Yosuke Nakazaki[1,4], Kei Kagii[1,4], Hiromi Imamura[2] & Yo-hei Watanabe[1,3]*

[1]Department of Biology, Faculty of Science and Engineering, Konan University, Kobe, Japan

[2]Department of Functional Biology, Graduate School of Biostudies, Kyoto University, Kyoto, Japan

[3]Institute for Integrative Neurobiology, Konan University, Kobe, Japan

[4]These authors contributed equally to this work.

Correspondence should be addressed to: Y.-h.W. (ywatanab@center.konan-u.ac.jp)

METHODS

Size exclusion chromatography analysis of *T*K-FRET. *T*K-FRET (0.1 mg·ml^{-1}) and its mutants dissolved in 50 mM MOPS-NaOH (pH 7.5), 150 mM KCl, and 5 mM $MgCl_2$ in the absence or presence of 2 mM ATP were incubated at 55 °C for 1 min. The mixture was then centrifuged at 21,000 *g* for 1 min. Aliquots (50 µl) of the solution were loaded on a TSKgel G-3000SWXL HPLC gel filtration column (Tosoh), equilibrated with the same beffer. The proteins were eluted at a flow rate 0.5 ml·min^{-1} at 55 °C. Absorbance was monitored at 280nm or 290 nm depending on the conditions, the absence or the presence of ATP, respectively.

Confirmation of aggregation of chaperone-fused EYFP. *T*ClpB and EYFP fusion proteins (6.0 µM monomer) in a mixture containing 50 mM MOPS-NaOH (pH 7.5), 150 mM KCl, 10 mM $MgCl_2$, and 5 mM TCEP was incubation at 80 °C for 15 min. The mixture was then centrifuged at 21,000 *g* for 10 min. The supernatant and the precipitated fractions were analyzed by the polyacrylamide gel (10%) electrophoresis in 0.1% sodium dodecylsulfate. The gel images were acquired by using ImageQuant LAS 4010 system (GE Healthcare). The supernatant fractions that were separately prepared in the same conditions were applied to a further centrifugation at 21,000 *g* for 1 min. An aliquot (15 µl) of the solution was loaded on a TSKgel SuperSW3000 HPLC gel filtration column (Tosoh) with guard column (Tosoh) equilibrated with 50 mM MOPS-NaOH (pH 7.5), 500 mM KCl, 5 mM $MgCl_2$, and 2 mM ADP. The proteins were eluted at a flow rate 0.35 ml·min^{-1} at room temperature. Absorbance was monitored at 290 nm. The proteins without 80 °C incubation were also applied to the same analysis.

Size exclusion chromatography analysis of the fusion proteins. *T*K-B, *T*K_NBD-B, *T*K_ΔSBDα-B, YFP-*T*B, YFP-*T*K_NBD-B, and their mutants (0.8-1 mg·ml^{-1}) dissolved in 50 mM MOPS-NaOH (pH 7.5), 150 mM KCl, and 5 mM $MgCl_2$ were incubated at 55 °C for 1 min in the presence of 2 mM ATP. The mixture was centrifuged at 21,000 *g* for 1 min. Aliquots (50 µg of protein) of the solution were loaded on a TSKgel SuperSW3000 HPLC gel filtration column with guard column, equilibrated with the same buffer. The proteins were eluted at a flow rate 0.35 ml·min^{-1} at 55 °C. Absorbance was monitored at 290 nm.

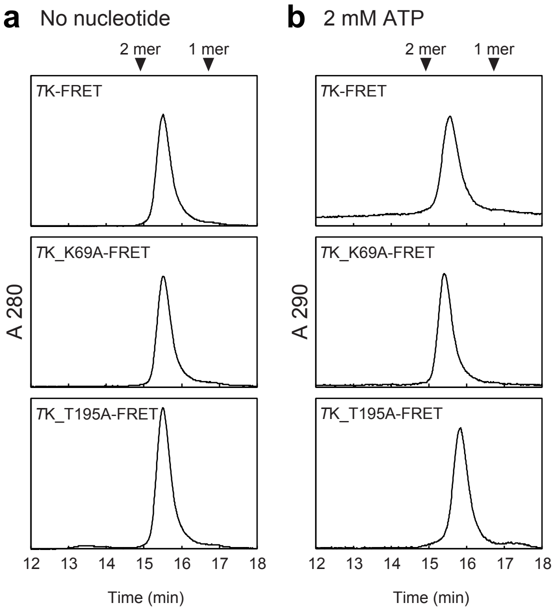

Hayashi *et al.* Fig. S1

Supplemental Figure 1 Size exclusion chromatography analysis of *T*K-FRETs. *T*K-FRET and its mutants (5 μg) were analyzed by a size exclusion chromatography in the absence (**a**) or the presence (**b**) of 2 mM ATP at 55 °C. The all *T*K-FRET variants showed almost the same elution profile in both conditions. The estimated molecular weight was between the monomer and the dimer of these proteins. Considering the fact that the shape of *T*K-FRETs was ellipsoidal, *T*K-FRETs were presented as monomers regardless of the presence of ATP. Though the ATP-induced dimerization of *E*DnaK was reported[1], at least in this experimental conditions, all the *T*K-FRETs did not cause dimerization that might disturbed the interpretation of FRET experiments. The calculated elution times corresponding to the monomer and the dimer of this protein were shown by black arrow.

1. Sarbeng, E.B. et al. A functional DnaK dimer is essential for the efficient interaction with Hsp40 heat shock protein. *J Biol Chem* **290**, 8849-62 (2015).

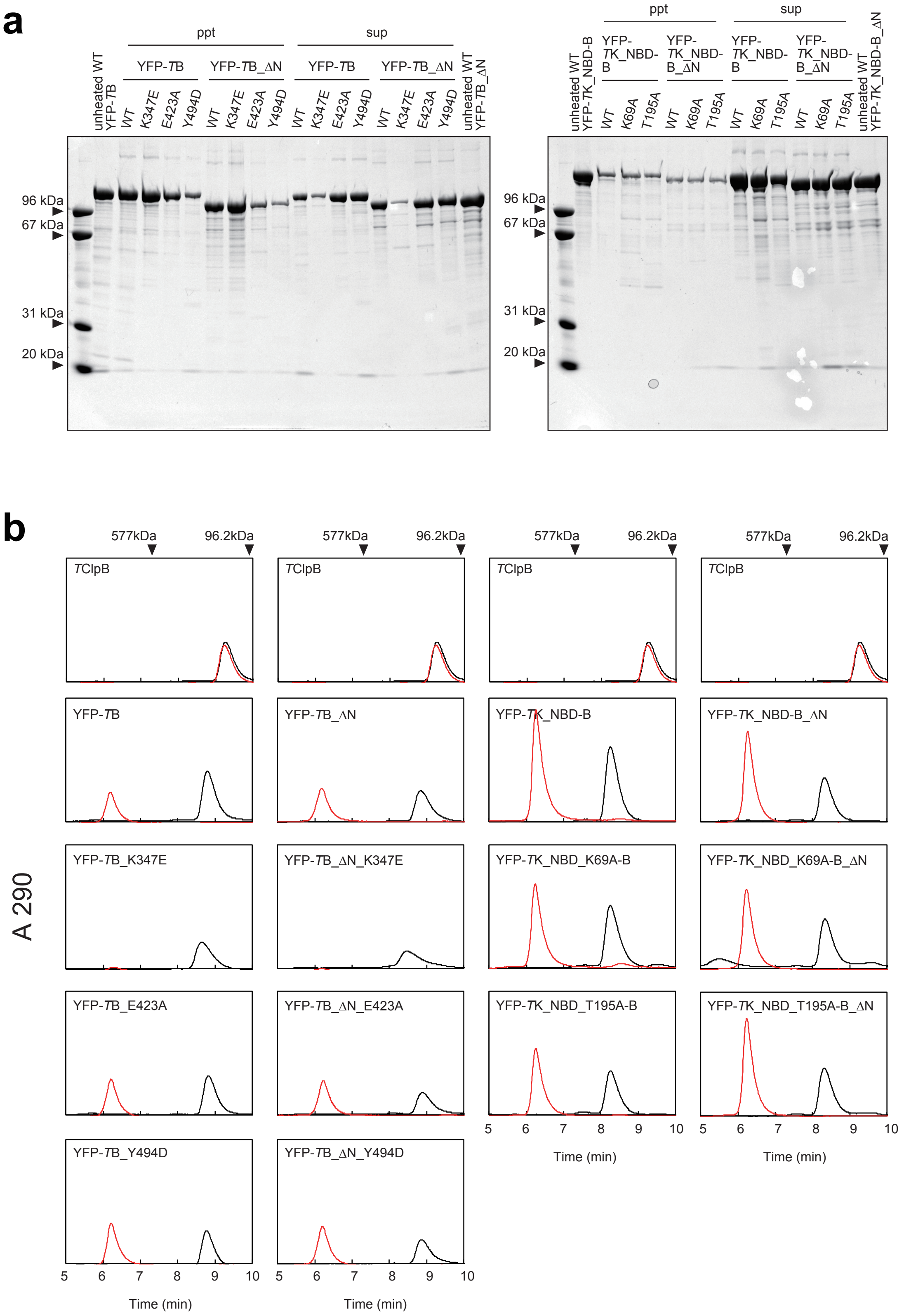

Hayashi *et al.* Fig. S2

Supplemental Figure 2 Confirmation of aggregation of the chaperone-fused EYFPs. (**a**) EYFP fusion proteins (6.0 μM monomers) were incubated at 80 °C for 15 min, and subsequently centrifuged. The supernatant (sup) and the precipitated (ppt) fractions were analyzed by SDS-PAGE. As the chaperones were derived from thermophilic bacteria, only the EYFP portion of these fusion proteins would be aggregated by the incubation at 80 °C for 15 min. Though, generally, the aggregated insoluble proteins are completely precipitated by the high speed centrifugation, the heat-treated fusion proteins were distributed to the supernatant and precipitated fractions in various ratio dependent on the fusion proteins. This might be caused by the differences of the intrinsic solubility of the chaperone portions. (**b**) To test the aggregation formation of EYFP portion, the supernatant fractions of heat-treated fusion proteins were analyzed by a size exclusion chromatography in the presence of 500 mM KCl and 2 mM ADP at room temperature. Untreated EYFP fusion proteins (black line) were eluted as monomers or dimers in this condition. However, all the supernatant fractions of heat-treated (at 80 °C for 15 min) EYFP fusion proteins (red line) were eluted at the position of higher order oligomers. Such a higher order oligomer was not observed in the case of the heat-treated non-fused *T*ClpB. These results indicated that most of all the EYFP portions were aggregated and tethering the subunits to each other. The calculated elution times corresponding to the monomer (96.2 kDa) and the hexamer (577 kDa) of *T*ClpB were shown by black arrow.

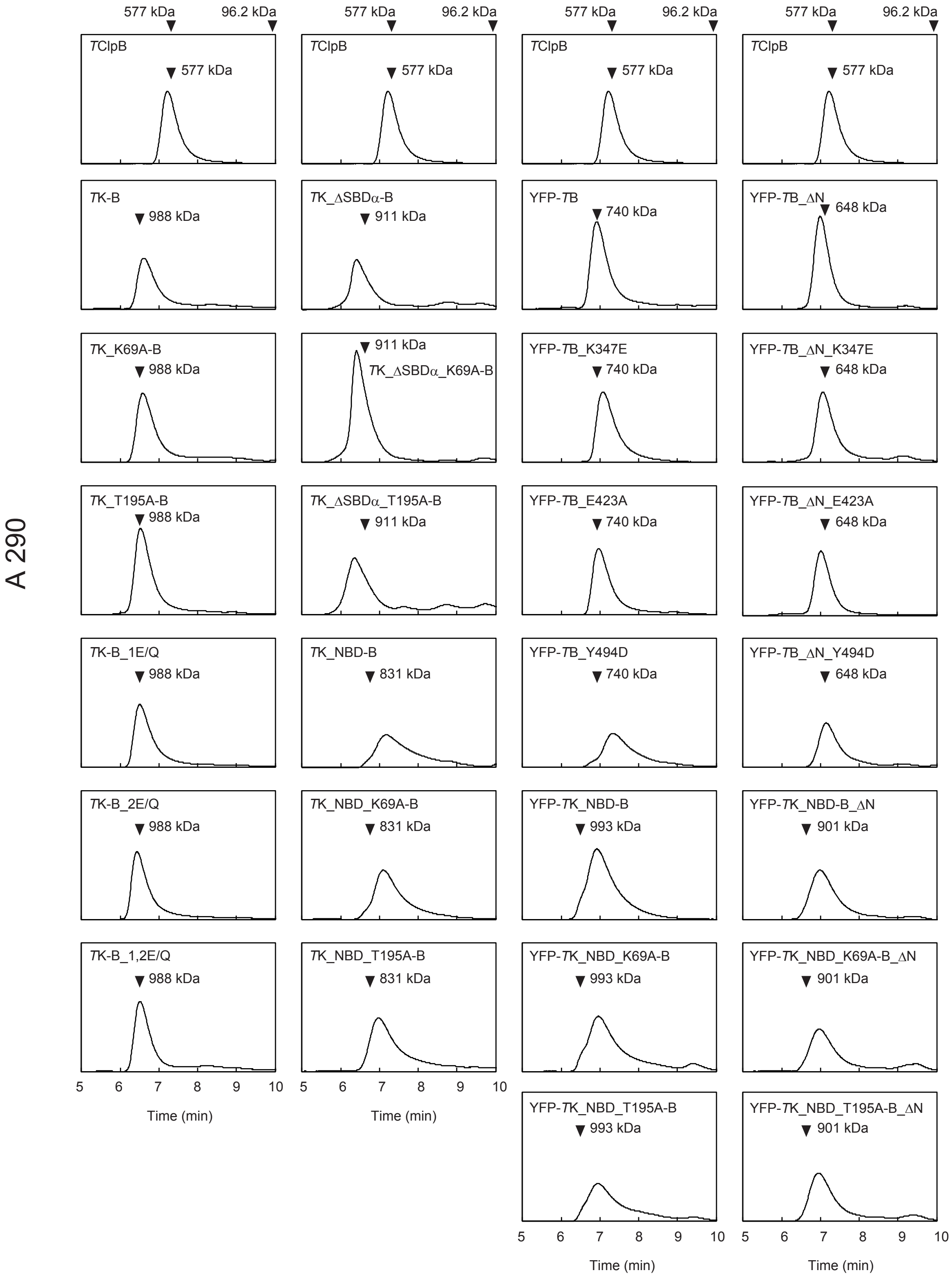

Hayashi *et al.* Fig. S3

Supplemental Figure 3 Size exclusion chromatography analysis of the fusion proteins. The all fusion proteins used here, *T*K-B, *T*K_NBD-B, *T*K_ΔSBDα-B, YFP-*T*B, YFP-*T*K_NBD-B, and their mutants (50 μg) were analyzed by a size exclusion chromatography in the presence of 2 mM ATP at 55 °C. In this condition, ClpB hexamer was stabilized. All these proteins were eluted as hexamer or close to it. Some fusion proteins having a high ATPase activity, such as *T*K_NBD-B, seemed slightly unstable, this might be caused by the oligomer destabilizing effect of the ADP produced by the hydrolysis of the surrounding ATP by the fusion proteins themselves. The calculated elution times corresponding to the hexamer of each fusion protein were shown by black arrow.
